# Hemodynamic and respiratory support in pulmonary embolism: a narrative review

**DOI:** 10.3389/fmed.2023.1123793

**Published:** 2023-06-02

**Authors:** Orlando Rubén Pérez-Nieto, Irene Gómez-Oropeza, Andrés Quintero-Leyra, Ashuin Kammar-García, Éder Iván Zamarrón-López, Maximiliano Soto-Estrada, Luis Antonio Morgado-Villaseñor, Héctor David Meza-Comparán

**Affiliations:** ^1^Unidad de Cuidados Intensivos, Hospital General San Juan del Río, Querétaro, Mexico; ^2^Department of Health Science, Universidad de las Américas Puebla, San Andrés Cholula, Puebla, Mexico; ^3^Coordinación Estatal de Capacitación, Mexican Red Cross, Estado de México, Mexico; ^4^Dirección de Investigación, Instituto Nacional de Geriatría, Mexico City, Mexico; ^5^Unidad de Cuidados Intensivos, Hospital MAC Tampico, Tampico, Tamaulipas, Mexico; ^6^Departamento de Emergencias, Hospital General de Zona 11 IMSS Delicias, Delicias, Chihuahua, Mexico; ^7^Unidad de Cuidados Intensivos, Hospital Christus Muguerza, Reynosa, Tamaulipas, Mexico

**Keywords:** pulmonary embolism, respiratory therapy, oxygen inhalation therapy, fluid therapy, diuretics, vasoconstrictor agents, vasodilator agents, extracorporeal membrane oxygenation

## Abstract

Pulmonary embolism is a common and potentially fatal disease, with a significant burden on health and survival. Right ventricular dysfunction and hemodynamic instability are considered two key determinants of mortality in pulmonary embolism, which can reach up to 65% in severe cases. Therefore, timely diagnosis and management are of paramount importance to ensure the best quality of care. However, hemodynamic and respiratory support, both major constituents of management in pulmonary embolism, associated with cardiogenic shock or cardiac arrest, have been given little attention in recent years, in favor of other novel advances such as systemic thrombolysis or direct oral anticoagulants. Moreover, it has been implied that current recommendations regarding this supportive care lack enough robustness, further complicating the problem. In this review, we critically discuss and summarize the current literature concerning the hemodynamic and respiratory support in pulmonary embolism, including fluid therapy, diuretics, pharmacological support with vasopressors, inotropes and vasodilators, oxygen therapy and ventilation, and mechanical circulatory support with veno-arterial extracorporeal membrane oxygenation and right ventricular assist devices, while also providing some insights into contemporary research gaps.

## Introduction

Pulmonary embolism (PE) remains a major cause of mortality worldwide, accounting for 50,000–300,000 deaths per year in the United States alone (1, 2). In Europe, it is responsible for another 300,000 deaths annually (3).

Mortality in PE is determined by several factors, in particular by the presence of right ventricular dysfunction (RVD) and/or hemodynamic instability (2, 4). High-risk PE makes up about 5% of the total number of PE presentations, although this may reflect that many patients die before admission, as demonstrated by postmortem studies (5). Altogether, in-hospital mortality due to PE ranges from 22.0 to 31.8%, increasing up to 65% in cases of cardiac arrest (4), with most deaths occurring within the first hour (6), which highlights the need for early recognition and treatment (7).

PE hinders both circulation and gas exchange (2). In this sense, supportive care (namely hemodynamic and respiratory support) plays a critical role in the comprehensive management of PE patients, especially in cases of respiratory failure and RVD (8), yet this very early management has been seldom studied (9) and given little attention in the recent literature, in favor of the novel advances concerning systemic thrombolysis, catheter-directed thrombolysis, direct oral anticoagulants, among others. Additionally, it has been pointed out that the available evidence concerning the use of supportive care in PE lacks sufficient robustness (8), which further complicates this problem.

For these reasons, in this paper, we aim to provide an up-to-date, critical review of the hemodynamic and respiratory support in PE, including fluid therapy, diuretics, pharmacological support with vasopressors, inotropes and vasodilators, oxygen therapy and ventilation, and mechanical circulatory support with veno-arterial extracorporeal membrane oxygenation (VA-ECMO) and right ventricular assist devices (RVAD). We will also review the mechanisms that lead to respiratory and circulatory failure in PE while providing a framework for risk stratification. Finally, we discuss the special considerations of advanced life support in the management of cardiac arrest caused by PE.

## Respiratory and circulatory failure in PE

Respiratory pathophysiology and subsequent gas exchange impairment, as well as RVD with accompanying left ventricle (LV) filling impairment via ventricular interdependence, play a pivotal role in the high risk of death from PE and are critical determinants of clinical severity and outcome (2, 10).

Vascular occlusion is the first phenomenon in PE. When emboli impact the pulmonary circulation, a local increase in the pulmonary arterial pressure occurs, resulting in pulmonary perfusion heterogeneity, with some hypoperfused areas –ultimately determining an increase in alveolar dead space– and other areas presenting blood overflow secondary to this regional pressure increment, leading to the heterogeneity in perfusion. This is followed by regional vasoconstriction induced by a neurologic reflex, together with endothelial and platelet cytokine release (10). Both mechanical occlusion and vasoconstriction converge, further reducing the vascular diameter. In short, zones of reduced flow in obstructed pulmonary arteries, in combination with zones of overflow in the capillary bed served by non-obstructed pulmonary vessels, lead to ventilation/perfusion (V/Q) mismatch, which contributes to hypoxemia in PE (2). In turn, hypoxemia is sensed by carotid chemoreceptors, and this stimulates the respiratory center, causing tachypnea, often accompanied by hypocapnia (10), which occurs in individuals with an intact respiratory drive (e.g., patients not under sedation).

In one-third of patients, shunting of venous blood into the systemic circulation through a patent foramen ovale may occur (11), caused by an inverted pressure gradient between the right atrium and left atrium, which may lead to severe hypoxemia and give rise to paradoxical embolization and stroke.

On the other hand, the sudden increase in pulmonary vascular resistance due to PE results in right ventricle (RV) dilation, an increase in RV wall tension, and a prolongation of RV contraction time into early diastole. This increased RV wall tension increases the local demand for oxygen, causing ischemia of the RV and decreased contractility, with a subsequent reduction in RV output (2, 10). This hampers the LV preload, which is additionally impaired by decreased LV distensibility as a consequence of a leftward shift of the interventricular septum (7). All in all, these mechanisms ultimately lead to a reduction of cardiac output (CO), cardiogenic shock, and death (2).

**Figure 1:**
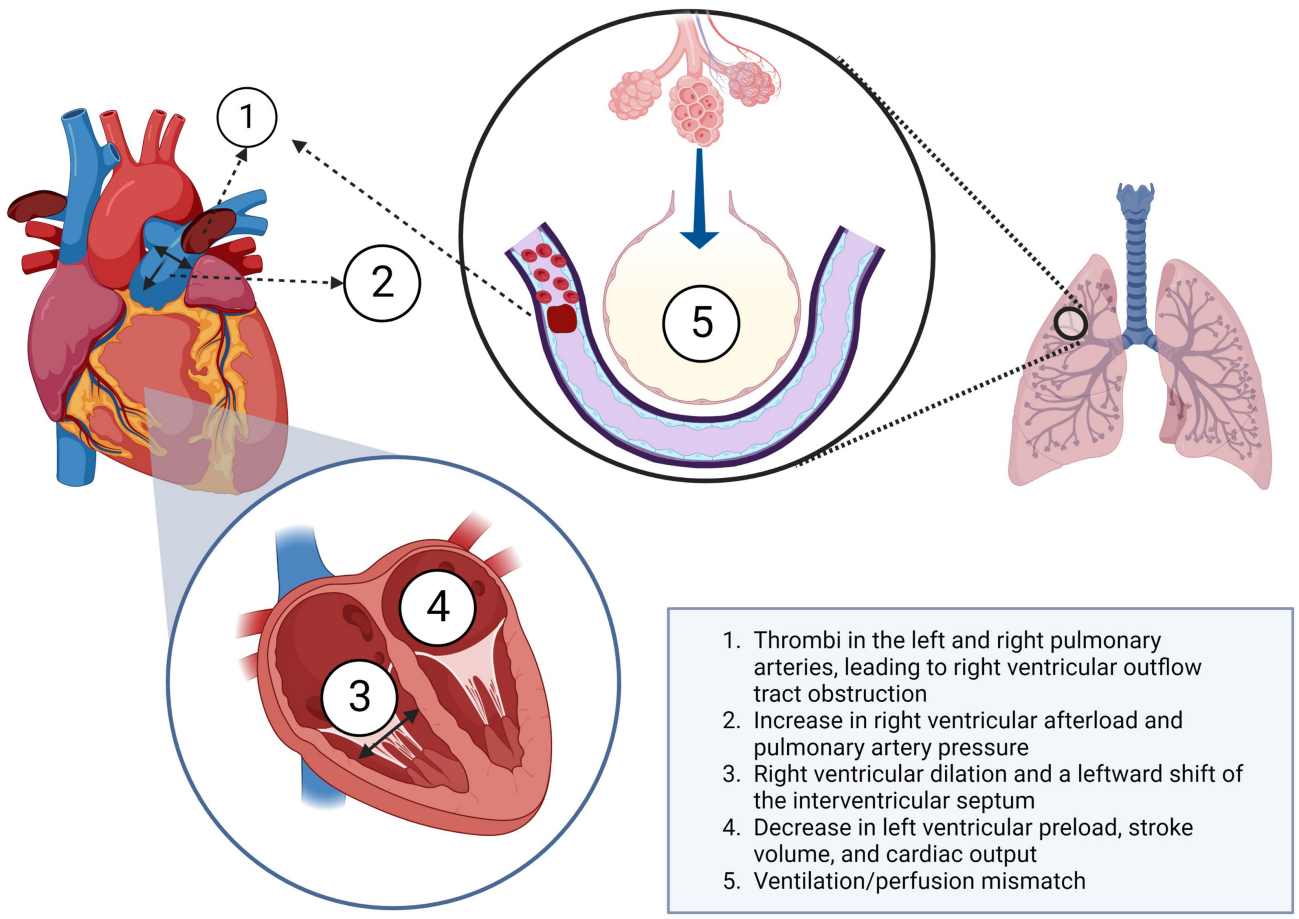
Pathophysiology of respiratory and circulatory failure in pulmonary embolism. Created with BioRender.com.

The key factors contributing to respiratory and circulatory failure in PE are shown in Figure 1.

## Risk stratification

Risk stratification allows for the deliverance of optimal treatment (5). Initially, it should be based on the identification of signs and symptoms of hemodynamic instability, which defines high-risk PE. Hemodynamic instability encompasses one of three main forms of clinical presentation: (1) cardiac arrest, (2) obstructive shock –defined as systolic blood pressure (BP) < 90 mmHg or vasopressors required to achieve a BP ≥90 mmHg despite adequate filling status, and end-organ hypoperfusion–, or (3) persistent hypotension –defined as systolic BP <90 mmHg or systolic BP drop ≥40 mmHg, lasting longer than 15 min and not caused by new-onset arrhythmia, hypovolemia, or sepsis–. High-risk PE warrants primary reperfusion treatment (in most cases, systemic thrombolysis) in conjunction with hemodynamic stabilization. Nevertheless, the absence of hemodynamic instability does not exclude the onset (and possibly progressing) of RVD, and thus a high PE-related mortality risk. Therefore, further risk stratification is recommended, since it has implications for prognosis, early discharge/hospitalization, and monitoring. In this regard, major advances have been achieved owing to the early mortality risk classification by the 2019 European Society of Cardiology (ESC) guidelines, which integrates bedside clinical scoring systems such as the Pulmonary Embolism Severity Index (PESI) and simplified PESI (sPESI), signs of RVD on imaging (whether on echocardiography or computed tomography pulmonary angiography), and cardiac biomarkers (such as cardiac troponins) (2, 4).

As a general rule, a PESI of class I-II or sPESI of 0 are reliable predictors of low-risk PE. This category is also marked by the absence of hemodynamic instability, signs of RVD, and elevated cardiac troponins. In this setting, guidelines suggest home treatment with anticoagulants when circumstances are adequate (12).

As opposed, patients in the intermediate-high-risk category display evidence of both RVD and elevated cardiac biomarkers, while the intermediate-low-risk category is defined by either presence of RVD or increased cardiac biomarkers (or none of them). Close in-hospital monitoring is advised in such cases to allow for the early detection of hemodynamic deterioration (2).

## Oxygen therapy and ventilation

In experimental PE models, oxygen therapy has been shown to reduce RV afterload and lower its mechanical work (13). Supplemental oxygen is indicated in patients with PE and arterial oxygen saturation (SaO_2_) <90%, starting with conventional devices such as low-flow nasal cannulas, standard face masks, or nonrebreather masks. However, if this fails, escalation of respiratory support may be warranted, including high-flow nasal cannula (HFNC) and mechanical ventilation (MV) –whether invasive or non-invasive– when necessary (2).

HFNC is capable of delivering 20–80 L/min (14) of a heated, humidified mixture of air and oxygen, and it has been shown to reduce reintubation rates and mortality in patients with acute hypoxemic respiratory failure (15). Additionally, HFNC reduces the work of breathing and respiratory rate (16) and increases the end-expiratory lung volume and pulmonary compliance (15). A systematic review and meta-analysis showed that, compared with conventional oxygen therapy, HFNC reduced the need for escalation of respiratory support, reduced dyspnea, and improved patient comfort (17). A recent observational study found a rapid improvement (as early as 1 h) in respiratory distress in PE patients using HFNC, in terms of oxygenation and respiratory rate. Furthermore, HFNC is superiorly tolerated compared to non-invasive ventilation (NIV), since it provides a high fraction of inspired oxygen (FiO_2_) and minimal essential positive end-expiratory pressure (PEEP) *via* nasal prongs (18). Therefore, when feasible, oxygen delivery through HFNC should be preferred (2).

Intubation and subsequent invasive mechanical ventilation (IMV) should be performed only if the patient is unable to tolerate NIV and after exhaustion of the above-described treatment modalities, given the detrimental effects of induction of anesthesia and positive-pressure ventilation on BP in PE patients with RVD. In this sense, anesthetic drugs more prone to cause hypotension, such as propofol, should be avoided. Therefore, etomidate, which is the drug of choice for induction in unstable patients, or ketamine, may be used in this setting unless contraindicated (2, 19). Indeed, when MV is required, care should be taken to limit its adverse hemodynamic effects. Notably, the positive intrathoracic pressure induced by MV may decrease venous return and worsen RVD in patients with shock. Hence, PEEP should be applied with caution and, if possible, should be aimed at 0 cmH_2_O (5, 7). Low tidal volumes (roughly 6 mL/kg lean body weight) should be used to maintain the end-inspiratory plateau pressure below 30 cmH_2_O (2, 7, 20), although these are ultimately expert opinions. It is essential to avoid mechanical ventilation as much as is viable since it increases hospital stay and the cost of care, and it is related to poor outcomes. Additionally, it is imperative to consider advanced treatments in patients with severe respiratory failure. In the Pulmonary Embolism Thrombolysis (PEITHO) trial, the authors compared the efficacy and safety of fibrinolytic therapy with a single-bolus injection of tenecteplase, in addition to standard anticoagulation therapy with heparin, versus placebo plus heparin, in normotensive patients with intermediate-risk PE. In subgroup analyses, patients who had a respiratory rate > 24 rpm (respirations per minute) achieved the primary efficacy outcome (a clinical composite of death from any cause or hemodynamic decompensation within 7 days after randomization) less frequently with the use of tenecteplase plus heparin, as opposed to patients in the placebo plus heparin arm, suggesting benefits for this intervention (21).

## Fluid therapy and diuretics

Intravenous (IV) fluids should be used with caution, since aggressive volume expansion may worsen RV function by causing mechanical overstretch/distension and/or inducing reflex mechanisms that further depress its contractility, ultimately leading to a reduction in systemic CO (5, 7). Thus, judging the appropriate amount of fluid administration is particularly difficult (22). In this regard, the 2019 ESC guidelines suggest a modest (≤500 mL) fluid challenge in patients with low central venous pressure (CVP), as it may increase the cardiac index in patients with PE, though this recommendation is based upon results of a small study conducted in the 1990s (2, 23). These guidelines also advocate for the guidance of volume loading through monitoring of CVP (e.g., by ultrasound imaging of the inferior vena cava). If signs of elevated CVP are present, further volume loading should be withheld. In practice, there is no reliable, well-validated standard for predicting volume responsiveness in acute RVD. Therefore, clinical judgment remains topmost, and every patient should be individually assessed (24).

On the flip side, a recent interest in the use of diuretics in PE has emerged. These drugs, which are capable of reducing RV preload, may be more appropriate than volume expansion in this setting, but they are commonly viewed as contraindicated because of the fear of depressing right ventricular function with accompanying abrupt loss of CO. One of the first major studies in this respect is Lim and colleagues’ randomized controlled trial (RCT), which aimed to compare the efficacy and safety of a single IV bolus of 80 mg furosemide against placebo in normotensive patients with intermediate-risk PE. In their study, a higher proportion of patients in the furosemide arm achieved the primary efficacy outcome (a composite of urine output >0.5 mL/kg/h, heart rate ≤ 110 bpm, systolic BP ≥100 mmHg and oxygen saturation ≥ 90%), compared with the placebo arm, with a relative risk (RR) of 1.30 (95% CI 1.04–1.61; *p* = 0.021), which means that furosemide increased the probability of achieving the primary efficacy outcome by 30% when compared to placebo. However, in their study, furosemide did not decrease *B*-type natriuretic peptide (BNP) or N-terminal pro *B*-type natriuretic peptide (NT-proBNP) concentrations, nor it changed right ventricular/left ventricular diameter ratio in echocardiography, as compared to placebo. Furthermore, furosemide increased creatinine levels and decreased systolic BP 24 h after randomization, suggesting that the chosen dose might have been excessive (25). One recent randomized, open-label trial explored the effects of diuretic therapy versus volume expansion in PE patients. The authors compared time to troponin normalization, time to BNP normalization, and changes in RV function through echocardiography, among intermediate-high-risk PE patients receiving either an IV bolus of 40 mg furosemide on admission (diuretic therapy arm), or a 500 mL 0.9% sodium chloride infusion delivered over 4 h, followed by a 1,000 mL infusion per day (volume expansion arm). Notably, troponin kinetics did not differ between the groups, although the time to complete BNP normalization was shorter in the diuretic therapy group, as was the time to 50% concentration decrease. In this study, the number of patients with decreased BNP concentrations at 12 h after randomization was also higher in the diuretic therapy arm. Furthermore, patients in this arm achieved a higher decrease in systolic pulmonary artery pressure and inferior vena cava diameter at 4 h after randomization, as measured by echocardiography, compared to patients in the volume expansion group. In conjunction, the findings of this study may reflect earlier RV function recovery with diuretic therapy in intermediate-high-risk PE patients (9).

It is important to remember that in patients with PE, the clinical problem is RVD and not systemic overload. The use of diuretics decreases preload, one of the ventricular function determinants, which may induce further hemodynamic deterioration. Until further studies are conducted, the choice between diuretic therapy and volume loading in PE patients must remain empirical (9).

## Pharmacological support with vasopressors, inotropes, and vasodilators

PE patients will oftentimes require vasopressor support in addition to the abovementioned measures to restore RV function and maintain coronary perfusion, in parallel with (or while waiting for) pharmacological, surgical, or interventional reperfusion treatments. Vasopressor support should be considered early in the resuscitation of patients with hypotension due to PE. On this basis, escalation of care to an intensive care unit (ICU) with close monitoring and multidisciplinary team care is required. Hence, transfer to a center that can provide this level of support must be considered if not available at the treating center (2, 4, 22, 26). Peripheral administration of vasopressors appears to be safe during the first 24 h. Furthermore, it reduces the risk of large vessel injury that can lead to bleeding whenever thrombolytics are being used (26). Norepinephrine (NE) is an alpha-adrenergic and beta-adrenergic drug that improves systemic pressure with a modest effect on inotropy and is also the vasopressor of choice in cardiogenic shock (24, 27). Though no direct clinical data are available on the effects of this drug in patients with PE (7), NE is currently recommended as the first-line vasopressor for patients with PE, at a dose of 0.2–1.0 μg/kg/min (2, 4), and it should be titrated to maintain a mean arterial pressure (MAP) >65 mmHg (5, 26). NE is preferred because it maintains coronary perfusion pressure and improves systemic vascular resistance (SVR), without increasing pulmonary vascular resistance (PVR) (2, 28). Nevertheless, at higher doses, there is the theoretical possibility of increasing PVR, but this seldom has a practical clinical impact. Vasopressin, another frequently suggested option, is a non-catecholamine vasoconstrictor that theoretically increases SVR without increasing PVR, though lack of robust data regarding its application in PE, minimal titratability, and absence of inotropic properties all limit its use. Adding vasopressin 0.04 U/min (as a second vasopressor) when NE dose >15 μg/min has been proposed (24, 28).

If CO remains low despite the options mentioned above, additional inotropic support may be required (28). Studies conclusively documenting the beneficial effect of inotropic agents in PE are rare (8). Dobutamine may increase cardiac index and decrease vascular resistance in PE patients (26, 29), though at the expense of systemic vasodilation, which can worsen hypotension if used alone (28). The 2019 ESC guidelines state that dobutamine may be considered for PE patients with a low cardiac index and normal BP, at a dose of 2–20 μg/kg/min (2). Nevertheless, an increased cardiac index above physiological values may worsen V/Q mismatch through additional redistribution of flow from partially obstructed to unobstructed vessels (7). Consequently, it is reasonable to use moderate doses of up to 10 μg/kg/min (8, 24). Data regarding the potential use of the inotrope/calcium sensitizer levosimendan in PE are also scarce (8, 28), yet it might be worth considering it in patients under beta-blockers (30). Preclinical studies suggest that this drug may restore RV-pulmonary arterial coupling by both an increase in RV contractility and a decrease in RV afterload (31), although no evidence of clinical benefit in PE is available as of this day (2). No studies conclusively prove the effectiveness of inotropes in PE patients. As observed in patients with cardiogenic shock secondary to left ventricular dysfunction, their potentially harmful effect is an unanswered question.

A few treatments have also been proposed, but they cannot be currently recommended due to the lack of conclusive data (5). In this sense, pulmonary vasoconstriction has long been explored as a specific target in PE (28), since it is widely considered a significant contributor to the increase of pulmonary artery pressure (PAP) and PVR in this context. Some of the pathways involved include the nitric oxide (NO)-soluble guanylate cyclase (sGC)-cyclic guanosine monophosphate (cGMP) pathway, the prostanoid pathway, and the endothelin (ET) pathway, to mention a few (32). Within the first category, inhaled nitric oxide (iNO) has recently gained some interest as a potential therapeutic agent, given its short half-life and limited systemic absorption (24). A 2015 systematic review of mostly case reports and case series showed improvement in either systemic or pulmonary pressures or oxygenation following iNO administration in PE patients, with an acceptable safety profile, although the authors raised concerns about possible reporting biases (33). More recently, an RCT compared the administration of iNO versus placebo in patients with intermediate-risk PE. Even though the study failed to demonstrate an improvement in the primary outcome (a composite of normal RV function on echocardiography and normal troponin T), secondary analyses suggested that iNO may increase the likelihood of resolving RV hypokinesis and dilation assessed by echocardiography. Additionally, no patient was discontinued from iNO for safety reasons and no serious adverse events were attributed to the study drug (34). On the other hand, the therapeutic potential of sildenafil, a specific PDE_5_ inhibitor (which acts on the NO-sGC-cGMP pathway), has also regained some interest. A recent explorative trial compared a single oral dose of 50 mg sildenafil versus placebo, to test if the former improved RV function in patients with intermediate-high-risk PE. Notably, sildenafil did not improve cardiac index compared to baseline. On top of that, sildenafil lowered MAP, which was not observed in the placebo arm (35). Concerning the prostanoid pathway, a small, single-blinded RCT compared the effects of IV epoprostenol, the pharmacological form of prostacyclin, versus placebo on echocardiographic and biochemical parameters in patients with PE with echocardiographic signs of RV overload. In this trial, epoprostenol did not have a significant effect on right ventricular end-diastolic diameter (RVED), systolic PAP, tricuspid annular plane systolic excursion (TAPSE), right ventricular fractional area change (RVFAC), serum cardiac troponin T and NT-proBNP, as compared to placebo (36). Lastly, the evidence concerning the use of vasodilators affecting the endothelin pathway in PE is limited, since there are only preclinical studies in existence, which show that ET receptor antagonists mostly lower mean pulmonary arterial pressure (mPAP) and PVR, as well as increase CO. Some other vasodilators such as hydralazine have not been investigated for over 20 years, neither in experimental PE models nor in patients with PE (32). At present day, no international guideline currently recommends using vasodilators during an acute PE event.

## Mechanical circulatory support with VA-ECMO and RVAD

In the setting of PE, both VA-ECMO and RVAD support the failing RV by halting the cycle of RV distention and ischemia, restoring hemodynamic stability, without any direct intervention on the clot burden. These mechanical circulatory support strategies may serve as a bridge to RV recovery with anticoagulation alone. They may also serve as a bridge for the decision to move along with active thrombus removal therapies, including surgical embolectomy or catheter-directed thrombolysis (37).

Indeed, the physiological rationale of VA-ECMO is attractive, since it seems to be ideal for breaking the commonly named “RV death spiral” (38). This supportive treatment decompresses the RV by redirecting RV venous return to the ECMO circuit, while also increasing perfusion by pumping oxygenated blood into the arterial system. This allows for RV and pulmonary artery (PA) decompression, with subsequent decreases in RV end-diastolic volume, RV end-diastolic pressure, and RV myocardial oxygen consumption, enabling RV contraction with minimal preload and afterload. Typically, the ECMO circuit provides between 4 to 6 L flow and has the advantage of being mobile, which means that it can be readily transported to the patient both in and out of the hospital. Once VA-ECMO is initiated, patients are usually rapidly stabilized, providing a window for physicians to decide on the next PE treatment (37, 38). According to the 2019 ESC guidelines, VA-ECMO may be helpful in patients with high-risk PE, and circulatory collapse or cardiac arrest (2). Nonetheless, current ECMO literature on patients with PE is restricted to nonrandomized, single-center case series and observational studies. In fact, to date, there is no RCT addressing the place of ECMO, with or without other reperfusion therapies, in the management of high-risk PE. Further complicating this issue, the available literature is vastly heterogeneous arising from differences in the presentation of patients, differences in hemodynamic parameters, and differences in the incidence of pre-ECMO cardiopulmonary resuscitation (CPR), among other factors, making the comparison between studies challenging (37, 38).

VA-ECMO plus anticoagulation alone results in definitive therapy in about 45% of patients, given that a substantial proportion of them may achieve RV recovery without additional reperfusion therapy (37). However, in terms of mortality, several retrospective studies have reported conflicting results concerning this approach. On the other hand, the VA-ECMO plus surgical embolectomy approach seems appealing even though there is limited data to support this combined strategy, with the largest study published so far reporting a mortality of 29% in the group treated with ECMO and surgical embolectomy. Finally, data regarding the use of catheter-directed thrombolysis in association with ECMO in PE patients is too scant, and further research is needed to assess the effectiveness and safety of this strategy (38).

The use of VA-ECMO is not without risks; major adverse events derived from this procedure include cannulation site bleeding/hematoma, ipsilateral leg ischemia, and acute kidney injury, to mention a few (37, 39, 40). In general, even with a high incidence of pre-ECMO CPR (which can be as high as 70–100%), mortality is roughly 30%, though some studies have estimated survival rates between 38 and 95%, again showcasing the high heterogeneity in the existing literature (37, 39). It has been pointed out that survival is an imperfect metric of VA-ECMO success since it is frequently confounded by several exposures before the initiation of ECMO such as CPR, failure of other treatments, and underlying medical conditions. In this sense, rather than survival, clinical and echocardiographic parameters of RV function may be better indicators of efficacy, while safety should be monitored with carefully adjudicated ECMO-specific complications (37). All in all, VA-ECMO outcomes will depend on the experience of the center, as well as proper patient selection (2).

RVADs, on the other hand, are peripherally inserted pumps that function by bypassing the RV through pumping of RV preload into the pulmonary circulation. In the context of PE, two major RVADs have emerged. The first one of them, the Impella RP (Abiomed, Inc., Danvers, Mass) is a percutaneous RVAD system that can be placed through the femoral veins with a 23F sheath. This device is subsequently guided into the left PA over a wire under fluoroscopic guidance. The pump inflow is placed in the inferior vena cava while the outflow is in the left PA, providing up to 4 L flow per minute. Although promising, the literature concerning this device is restricted to various case reports, thus impeding drawing definite conclusions regarding its safety and efficacy in PE. The other device, the Protek Duo system (LivaNova, London, UK) is inserted through the right internal jugular vein and into the right PA under fluoroscopic guidance. The proximal port is located in the right atria and the distal port is in the right PA. This device is subsequently attached to an extracorporeal pump, currently being the only percutaneous RVAD capable of oxygenating. In a similar fashion to the Impella RP, the experience with the Protek Duo is restricted to case reports, and more research is needed to draw conclusions regarding its utility in PE. Potential pitfalls of these devices include lack of availability in some institutions and the need for fluoroscopy for device implantation (37, 41).

## Advanced life support in cardiac arrest caused by PE

PE is a part of the differential diagnosis of cardiac arrest with non-shockable rhythm. Current guidelines for advanced life support should be stuck to in cardiac arrest presumably stemming from PE (2), though there are some considerations to bear in mind in this situation, which we will carefully discuss. Concerning cardiac arrest prevention, the 2021 European Resuscitation Council Guidelines recommend following the ABCDE approach. First of all, on the *airway*, these guidelines state that high-flow oxygen therapy should be initiated in patients with life-threatening hypoxia. Within *breathing*, the guidelines advocate for considering PE in all patients with sudden onset of progressive dyspnea and absence of known pulmonary diseases (though the exclusion of pneumothorax and anaphylaxis is always necessary). On *circulation*, it is critical to identify hemodynamic instability and high-risk PE, plus obtaining a 12-lead ECG (making sure of excluding acute coronary syndrome, while also looking for signs of RV strain). The guidelines also suggest performing bedside echocardiography and initiating anticoagulation therapy (heparin 80 IU/kg IV) during diagnosis, unless signs of bleeding or absolute contraindications are present. PE diagnosis should be confirmed with computed tomographic pulmonary angiography (CTPA). Of note, these guidelines also advise setting up a multidisciplinary team for decision-making on the management of high-risk PE, if feasible. Rescue thrombolytic therapy should also be given in rapidly worsening patients; alternatives in this situation include surgical embolectomy or catheter-directed treatment. Within *exposure*, obtaining information about past medical history, predisposing risk factors, and medications that may support the diagnosis of PE is necessary, such as a history of previous PE or deep venous thrombosis (DVT), surgery or immobilization within the past 4 weeks, active cancer, clinical signs of DVT, oral contraceptives use or hormone replacement therapy (HRT), long-distance flights, among others. Regarding cardiac arrest management, the guidelines emphasize that cardiac arrest usually presents as pulseless electrical activity (PEA). They also highlight that, although a non-specific sign, low EtCO_2_ readings (<1.7 kPa/13 mmHg) while performing high-quality chest compressions may support a diagnosis of PE. Emergency echocardiography performed by a qualified sonographer should also be considered as an additional diagnostic tool in this context. These guidelines suggest administering thrombolytic drugs for cardiac arrest when PE is the suspected cause and, after administration, they recommend continuing CPR attempts for at least 60 to 90 min before termination. The use of thrombolytic drugs, surgical embolectomy, or percutaneous mechanical thrombectomy is advised when PE is the known cause of cardiac arrest. Finally, the guidelines recommend considering extracorporeal CPR (ECPR) as rescue therapy for selected patients with cardiac arrest in whom conventional CPR is failing, if feasible and according to local resources (42).

## Conclusion

**Figure 2:**
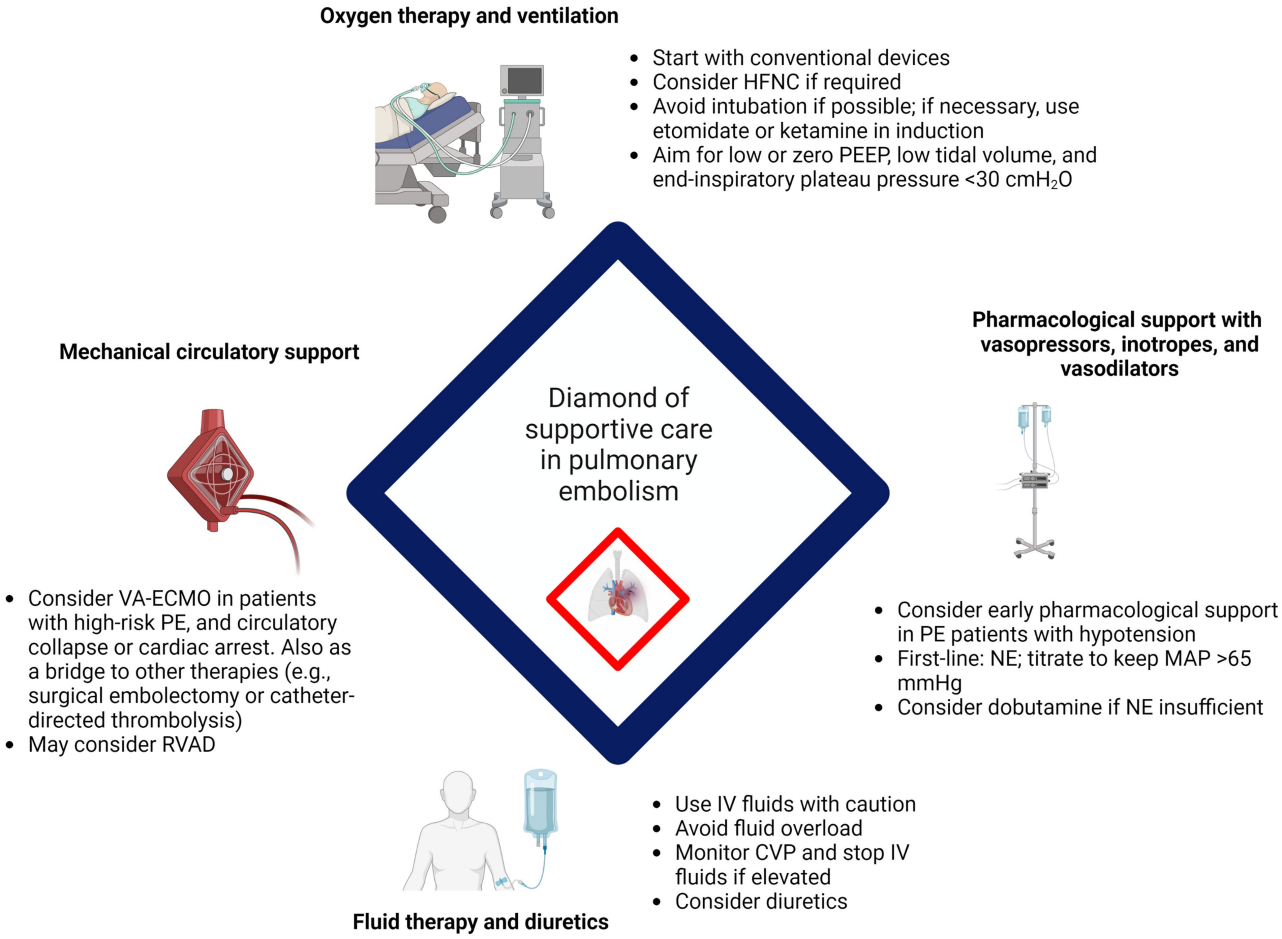
Diamond of supportive care in pulmonary embolism. Created with BioRender.com. Abbreviations: CVP, central venous pressure; HFNC, high-flow nasal cannula; IV, intravenous; MAP, mean arterial pressure; NE, norepinephrine; PE, pulmonary embolism; PEEP, positive end-expiratory pressure; RVAD, right ventricular assist device; VA-ECMO, veno-arterial extracorporeal membrane oxygenation.

The hemodynamic and respiratory support in PE is particularly complex and heterogenous but can be summarized in what we call the *diamond of supportive care in pulmonary embolism* (Figure 2). A cautionary approach is advised, since the harms may sometimes outweigh the benefits if used inappropriately. Current recommendations are fundamentally based upon studies with low levels of evidence, most of them being expert opinions, preclinical studies, and observational studies. Although some progress has been made, more high-quality research is needed to better inform clinical decisions concerning supportive therapy in PE. Hopefully, in this review, we may have sparked some interest in this research area within the ever-evolving field of PE management.

## Author contributions

OP-N: conceptualization. HM-C: investigation, writing - original draft, and writing - review and editing. AQ-L: investigation and writing - original draft. IG-O: investigation and writing - original draft. AK-G: methodology. ÉZ-L and LM-V: supervision. MS-E: visualization. All authors contributed to the article and approved the submitted version.

## Conflict of interest

The authors declare that the research was conducted in the absence of any commercial or financial relationships that could be construed as a potential conflict of interest.

## Publisher’s note

All claims expressed in this article are solely those of the authors and do not necessarily represent those of their affiliated organizations, or those of the publisher, the editors and the reviewers. Any product that may be evaluated in this article, or claim that may be made by its manufacturer, is not guaranteed or endorsed by the publisher.
